# Functional Analysis of *Cucumis melo CmXTH11* in Regulating Drought Stress Tolerance in *Arabidopsis thaliana*

**DOI:** 10.3390/ijms252011031

**Published:** 2024-10-14

**Authors:** Shiwen Zhao, Qianqian Cao, Lei Li, Wenqin Zhang, Yongjun Wu, Zhenchao Yang

**Affiliations:** 1 Key Laboratory of Northwest Facility Horticulture Engineering of Ministry of Agriculture and Rural Affairs, College of Horticulture, Northwest A & F University, Yangling 712000, China; a17633757139@163.com (S.Z.); caoqianqian2022@163.com (Q.C.); neilor777@163.com (L.L.); zwqhkhk@163.com (W.Z.); 2 College of Life Sciences, Northwest A & F University, Yangling 712000, China

**Keywords:** *CmXTH11*, water stress, drought tolerance, oxidative stress, antioxidant enzymes

## Abstract

The *CmXTH11* gene, a member of the *XTH* (xyloglucan endotransglycosylase/hydrolase) family, plays a crucial role in plant responses to environmental stress. In this study, we heterologously expressed the melon gene *CmXTH11* in *Arabidopsis* to generate overexpressing transgenic lines, thereby elucidating the regulatory role of *CmXTH11* in water stress tolerance. Using these lines of *CmXTH11* (OE1 and OE2) and wild-type (WT) *Arabidopsis* as experimental materials, we applied water stress treatments (including osmotic stress and soil drought) and rewatering treatments to investigate the response mechanisms of melon *CmXTH11* in *Arabidopsis* under drought stress from a physiological and biochemical perspective. Overexpression of *CmXTH11* significantly improved root growth under water stress conditions. The OE lines exhibited longer roots and a higher number of lateral roots compared to WT plants. The enhanced root system contributed to better water uptake and retention. Under osmotic and drought stress, the OE lines showed improved survival rates and less wilting compared to WT plants. Biochemical analyses revealed that *CmXTH11* overexpression led to lower levels of malondialdehyde (MDA) and reduced electrolyte leakage, indicating decreased oxidative damage. The activities of antioxidant enzymes, including superoxide dismutase (SOD), catalase (CAT), and peroxidase (POD), were significantly higher in OE lines, suggesting enhanced oxidative stress tolerance. The *CmXTH11* gene positively regulates water stress tolerance in *Arabidopsis* by enhancing root growth, improving water uptake, and reducing oxidative damage. Overexpression of *CmXTH11* increases the activities of antioxidant enzymes, thereby mitigating oxidative stress and maintaining cellular integrity under water deficit conditions. These findings suggest that *CmXTH11* is a potential candidate for genetic improvement of drought resistance in crops.

## 1. Introduction

Water deficit is one of the most critical factors influencing plant growth and development [1]. In drought-stressed environments, plants can mitigate this stress by modifying their morphological structures and enhancing physiological processes, such as metabolism [2]. The cell wall serves as a “boundary” between the cell and the external environment, capable of sensing stress signals and activating various signaling pathways, such as ABA-dependent and -independent pathways, calcium ion (Ca^2+^) signaling pathways, and reactive oxygen species (ROS) pathways [3]. This signaling cascade regulates the expression of key enzymes involved in synthesizing cell wall components at multiple levels, including transcription and translation, enabling plants to manage stress effectively [4,5,6].

Plant cell walls are primarily composed of cellulose, hemicellulose, and pectin, with xyloglucan being the predominant hemicellulosic polysaccharide found in primary plant cell walls [7]. Xyloglucan endotransglucosylase/hydrolase (*XTH*) plays a crucial role in modifying the structure of lignin–xyloglucan by catalyzing the cleavage and recombination of xyloglucan, which helps maintain the elasticity and strength of the cell wall [8]. Furthermore, *XTH*s are involved in various physiological and biological processes, including plant growth and stress resistance [9,10]. *XTH*s are widely distributed across plant species. For example, Studies have identified 33 *XTH* gene members in *Arabidopsis* [11], 29 in rice [12], and 25 in tomato [13].

Research indicates that *XTH*s play multiple roles in plant stress responses and are crucial for enhancing plant stress tolerance [14]. For instance, overexpression of the *CaXTH3* gene in pepper and *Arabidopsis* has been shown to improve drought resistance in transgenic plants [15]. Similarly, transferring *DkXTH1* from persimmon into *Arabidopsis* enhanced its tolerance to salt and drought stress [16]. Under aluminum stress conditions, *AtXTH31* participates in cell wall modification and cell elongation by regulating XET activity [17]. Furthermore, studies suggest that *AtXTH31* may also influence aluminum tolerance by modulating xyloglucan levels and its interaction with aluminum [18].

Melon (*Cucumis melo* L.) is an annual herbaceous plant belonging to the Cucurbitaceae family; it is extensively cultivated in Asia as a fruit and vegetable crop [19]. It has a shallow root system and large, thin leaves, which, combined with its preference for warm conditions, result in high water demand and relatively low stress tolerance. Our previous research identified and analyzed the expression of the *XTH* gene family in melon, revealing that *XTH*s respond to drought stress [20]. In this study, we focused on the *CmXTH11* gene, selecting it for overexpression in *Arabidopsis*. We subjected various genotypes to different water stress treatments, including osmotic stress, soil drought, and subsequent rehydration, to explore the role of *CmXTH11* in plant water stress tolerance. This research will significantly enhance our understanding of stress resistance mechanisms in melon.

## 2. Results

### 2.1. CmXTH11 Gene Structure and Evolutionary Analysis

#### 2.1.1. *CmXTH11* Protein Structure and Physicochemical Properties Analysis

In the melon genome database, the gene identified as LOC103503575 is named *CmXTH11* based on its chromosomal location. Analysis of the *CmXTH11* protein’s physicochemical properties revealed that the protein has a molecular mass of 40,244.39 Da and a molecular formula of C_1809_H_2717_N_497_O_521_S_15_. The protein consists of 348 amino acids, with an isoelectric point (pI) of 8.72, indicating that it is slightly basic. The average hydrophobicity coefficient is −0.475, suggesting that CmXTH11 is likely a hydrophilic protein.

**Figure 1:**
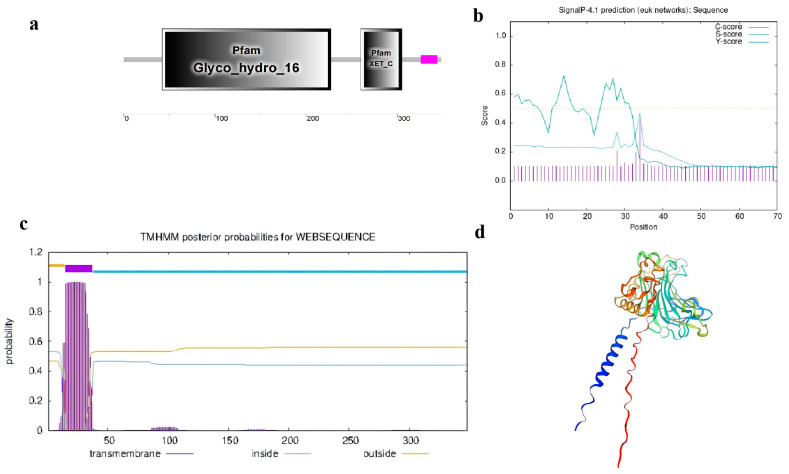
*CmXTH11* protein structure and physicochemical properties analysis. (**a**) Conserved domains of *CmXTH11* protein; (**b**) signal peptide domain analysis of *CmXTH11* protein; (**c**) transmembrane domain analysis of *CmXTH11* protein; (**d**) SWISS modeling of *CmXTH11*.

Domain analysis of *CmXTH11* using SMART (http://smart.embl-heidelberg.de/ (accessed on 10 September 2024)) identified two functional domains: the Glyco_hydro_16 domain and the XET_C domain (Figure 1a). Signal peptide analysis of *CmXTH11* indicated an S-score of 0.729 and a C-score of 0.430. A peak was observed at only one site, with a Y-score of 0.471, an S-mean between 1 and 33 amino acids with an average of 0.525, and a D-value of 0.493, suggesting that *CmXTH11* does not possess a signal peptide (Figure 1b). Additionally, TMHMM software 2.0 predicted that *CmXTH11* contains a transmembrane domain in *CmXTH11*, located at the N-terminal of the protein (Figure 1c). Using the SWISS-MODEL website (https://swissmodel.expasy.org/ (accessed on 10 September 2024)) to predict the tertiary structure of the amino acids encoded by *CmXTH11* (Figure 1d), the results indicate that the *CmXTH11* protein exhibits xyloglucan endotransglucosylase/hydrolase activity.

#### 2.1.2. *CmXTH11* Gene Expression Characteristics Analysis

**Figure 2:**
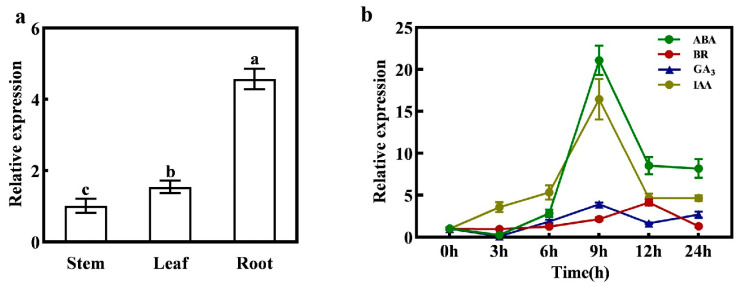
*CmXTH11* expression analysis. (**a**) Tissue-specific expression analysis of *CmXTH11*; (**b**) expression levels of *CmXTH11* under different hormone treatments. Note: Data in the same column with different letters indicate significant differences (*p* < 0.05).

The qPCR analysis of *CmXTH11* gene expression in different tissues of melon seedlings with expanded cotyledons is shown in Figure 2. The results reveal that *CmXTH11* is most abundantly expressed in roots, followed by leaves and stems, with significant differences observed among these tissues. The expression level in roots is markedly higher than that in stems and leaves (Figure 2a).

To investigate the response of *CmXTH11* to various plant growth regulators, qRT-PCR was employed to measure the gene’s expression changes under different treatments. The results demonstrated significant variations in *CmXTH11* expression across the different hormonal treatments (Figure 2b). Specifically, under ABA and NAA treatments, *CmXTH11* expression peaked at 9 h, reaching levels 20 times higher than at 0 h. For GA3 treatment, *CmXTH11* also reached its highest level at 9 h, though the increase was less pronounced compared to ABA and NAA. Under BR treatment, *CmXTH11* expression peaked at 12 h, showing a fourfold increase compared to 0 h.

#### 2.1.3. Evolutionary Analysis of *CmXTH11* Protein

**Figure 3:**
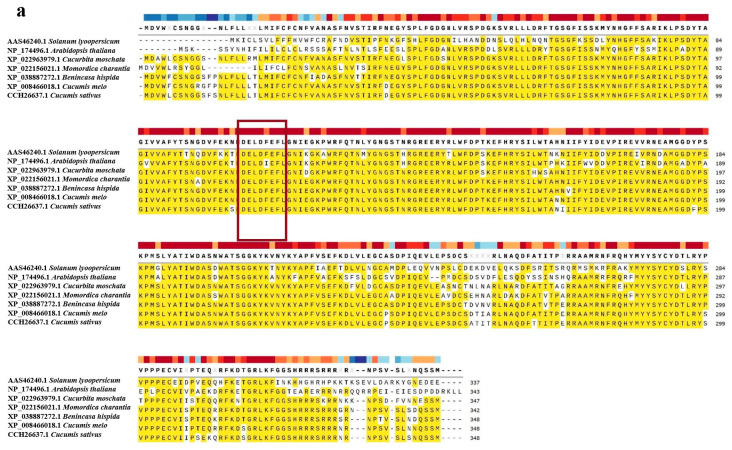

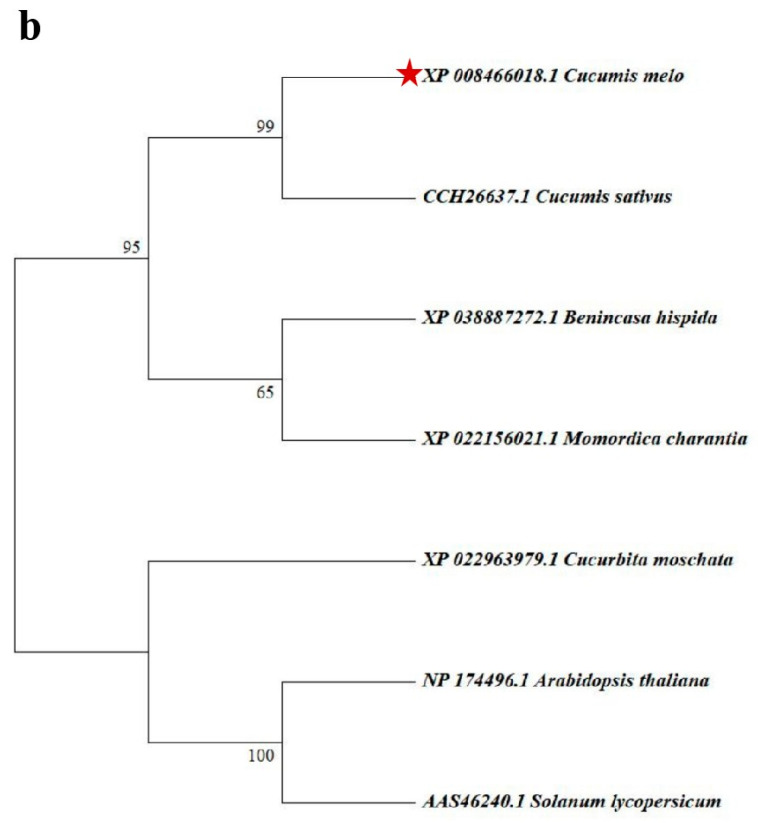
Analysis of *CmXTH11* amino acid sequences and phylogenetic tree construction. (**a**) Amino acid sequence alignment: Comparative analysis of the amino acid sequences of *CmXTH11* with *XTH* proteins from six different plant species; (**b**) phylogenetic tree analysis: Phylogenetic tree showing the evolutionary relationships between *CmXTH11* and *XTH* proteins from various plant species.

The protein sequences of *CmXTH11* were compared with homologous sequences from six plant species (Table 1): Winter melon (*Benincasa hispida*), bitter melon (*Momordica charantia*), pumpkin (*Cucurbita moschata*), *Arabidopsis thaliana*, cucumber (*Cucumis sativus*), and tomato (*Solanum lycopersicum*). The sequence alignment revealed that the homology between these sequences and *CmXTH11* ranged from 68.73% to 95.98% (Figure 3a). Analysis indicated that all these protein sequences contain a highly conserved catalytic domain, DEIDFEFLG, characteristic of xyloglucan endotransglucosylase (XET).

Phylogenetic tree analysis was performed to investigate the evolutionary relationships between *CmXTH11* and the homologous proteins from the six plant species. *CmXTH11* is most closely related to the *CsXTH* from cucumber (Figure 3b).

### 2.2. Confirmation of Transgenic Arabidopsis Lines

#### 2.2.1. Generation of Transgenic Plants

**Figure 4:**
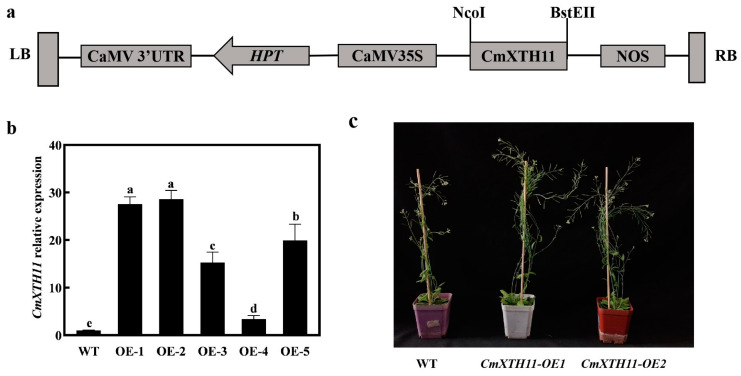
Confirmation of transgenic *Arabidopsis* lines. (**a**) Construction of the pCAMBIA1303-CaMV35S-*CmXTH11* expression vector; (**b**) expression levels of *CmXTH11* in 4-week-old *Arabidopsis* leaves of WT and two transgenic lines (OE1 and OE2); (**c**) phenotypic comparison of 8-week-old WT and *CmXTH11-OE* lines under normal growth conditions. Note: Data in the same column with different letters indicate significant differences (*p* < 0.05).

To investigate the potential functions of the *CmXTH11* gene, its coding sequence was inserted into the pCAMBIA1303 vector (Figure 4a) and transformed into *Arabidopsis* using the floral dip method. A total of five independent transgenic lines were obtained and confirmed through PCR analysis. To determine the expression levels of *CmXTH11*, two T3 homozygous lines with the highest relative expression levels (OE1 and OE2) were selected for further experiments (Figure 4b). Under normal growth conditions, the transgenic plants exhibited a significantly high number of inflorescences compared to the wild-type (WT) plants (Figure 4c).

#### 2.2.2. Analysis of Root Growth in Transgenic Plants

**Figure 5:**
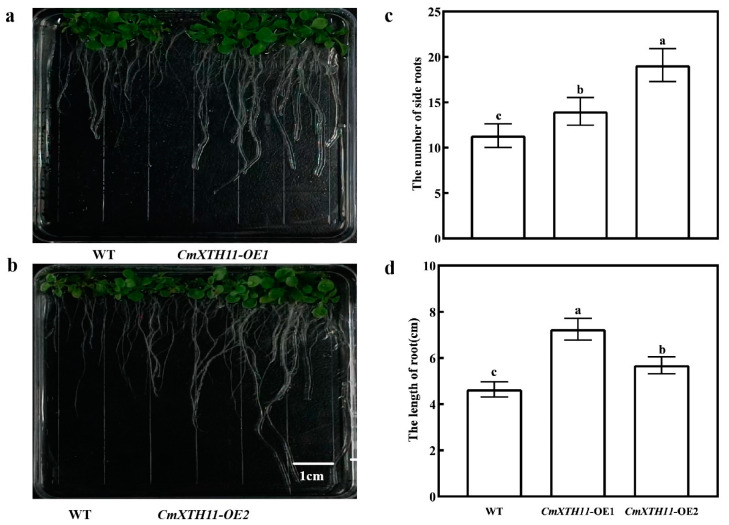
Analysis of root growth in WT and OE *Arabidopsis* lines. (**a**,**b**) Phenotypic images of root growth in WT and OE lines grown on 1/2 MS medium; (**c**) number of lateral roots in WT and OE lines grown on 1/2 MS medium; (**d**) root length of WT and OE lines grown on 1/2 MS medium. Note: Data in the same column with different letters indicate significant differences (*p* < 0.05).

The transgenic lines showed significantly longer primary roots than WT plants, with OE-2 and OE-1 being 22.52% and 56.13% longer, respectively (Figure 5a,b,d). Additionally, OE lines had more lateral roots, with OE-2 and OE-1 having 1.68 and 1.24 times the number of WT roots, respectively (Figure 5c).

### 2.3. Seedling Growth Analysis of Overexpressing CmXTH11 Arabidopsis

**Figure 6:**
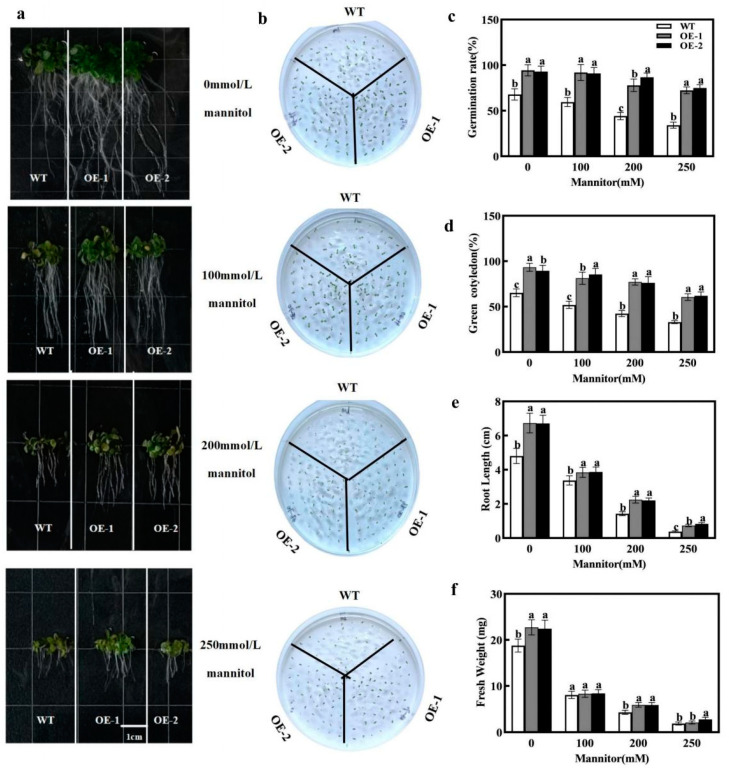
The impact of drought stress on seed germination in WT and *CmXTH11* overexpressing *Arabidopsis*. (**a**) Phenotypic images of root lengths of WT and transgenic lines on 1/2 MS medium containing 0, 100, 200, and 250 mM mannitol after 10 days of cultivation. (**b**) Phenotypic images of seed germination under the same conditions. (**c**) Seed germination rates under different mannitol concentrations. (**d**) Number of green leaves. (**e**) Root lengths of seeds grown under different mannitol concentrations. (**f**) Fresh weight of seeds grown under different mannitol concentrations. Note: Data in the same column with different letters indicate significant differences (*p* < 0.05).

To investigate the differences in osmotic stress tolerance between wild-type (WT) and overexpressing *CmXTH11 Arabidopsis* (OE1 and OE2) lines, we compared seed germination and root growth under osmotic stress conditions. Under 0 mmol/L mannitol conditions, the germination rate and root length of the transgenic lines were significantly higher than those of WT. At 100 mmol/L mannitol treatment, a noticeable difference in germination rates emerged, favoring the transgenic lines. Under 200 mmol/L mannitol stress, the germination rates of OE1 and OE2 remained above 75%, while that of WT was only 44.16%. When the mannitol concentration reached 250 mmol/L, the survival rate of all lines decreased; however, the survival rates of transgenic lines OE1 and OE2 were still higher than that of WT, with both exceeding 70% (Figure 6c).

After stress treatment, the root length and fresh weight of the transgenic lines decreased significantly compared to the non-stressed conditions, but both OE1 and OE2 lines were significantly higher than the WT. Notably, under 100 mmol/L, 200 mmol/L, and 250 mmol/L mannitol stress, the root lengths of OE2 and OE1 were 1.14, 1.5, and 2 times greater than that of WT plants, respectively (Figure 6e). At a 100 mmol/L mannitol concentration, no significant differences in fresh weight were observed among the three lines. However, under 200 mmol/L mannitol stress, the fresh weight of the OE-2 and OE-1 lines increased by 37.79% compared to that of the WT. Under 250 mmol/L mannitol stress, the fresh weight of the OE-2 and OE-1 lines increased by 47.87% and 12.23%, respectively, relative to that of the WT (Figure 6f). In summary, overexpression of *CmXTH11* alleviated the inhibitory effects of osmotic stress on *Arabidopsis* growth and significantly enhanced drought resistance.

### 2.4. Overexpression of CmXTH11 Gene Enhances Drought Resistance in Arabidopsis Seedlings

**Figure 7:**
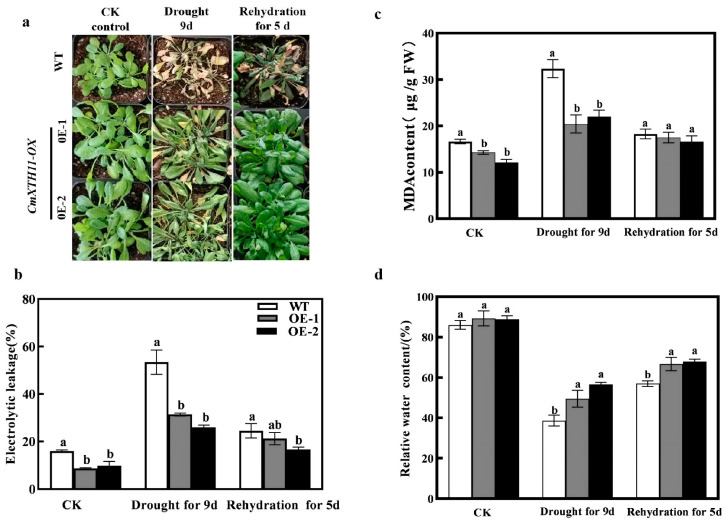
*CmXTH11* gene enhances drought resistance in *Arabidopsis*. (**a**) Phenotypes of WT and *CmXTH11* overexpressing lines (OE1, OE2) under normal growth conditions, after 9 days of drought stress, and after 5 days of rewatering; (**b**–**d**): malondialdehyde (MDA) content (**b**), relative electrolyte conductivity (REC) (**c**), and relative water content (RWC) (**d**) in WT and *CmXTH11*-OE lines under normal growth conditions, after 9 days of drought stress, and after 5 days of rewatering. Note: Data in the same column with different letters indicate significant differences (*p* < 0.05).

To further investigate the impact of the *CmXTH11* gene on water stress, we conducted drought and rewatering experiments using soil-grown seedlings. Under normal conditions, the leaves of overexpressing lines (OE1 and OE2) are larger than those of WT plants, although there are no significant differences in leaf status (Figure 7a). After 9 days of drought stress, WT plants exhibited more severe damage than the overexpressing lines, showing noticeable wilting and yellowing of the leaves. After 5 days of rewatering, the phenotypes of OE2 and OE1 plants recovered more substantially than WT plants.

The results (Figure 7c) show that under normal growth conditions, the malondialdehyde (MDA) content in the transgenic lines OE1 and OE2 is lower than in the wild-type (WT). After 9 days of drought stress, the MDA content in WT significantly increased significantly, reaching twice the level under normal conditions. However, the MDA content in the transgenic lines remained significantly lower than that in WT plants. WT plants reached 32.36 µg/g, while the levels in OE-1 and OE-2 were only 20.43 µg/g and 22.04 µg/g, respectively. After rewatering, there were no significant differences among the three groups. The relative electrolyte conductivity (REC) and MDA content exhibited similar trends (Figure 7b). Under normal conditions, there were no differences in relative water content (RWC) between WT and transgenic lines (Figure 7d). However, after 9 days of drought stress, the RWC in WT was significantly lower compared to the transgenic lines, with OE-1 and OE-2 showing increases of 28.06% and 46.33%, respectively (Figure 7d). After 5 days of rewatering, the RWC of the OE lines remained higher than that of the WT line.

### 2.5. Overexpression of CmXTH11 Gene Enhances Drought Stress Tolerance by Participating in ROS Scavenging

**Figure 8:**
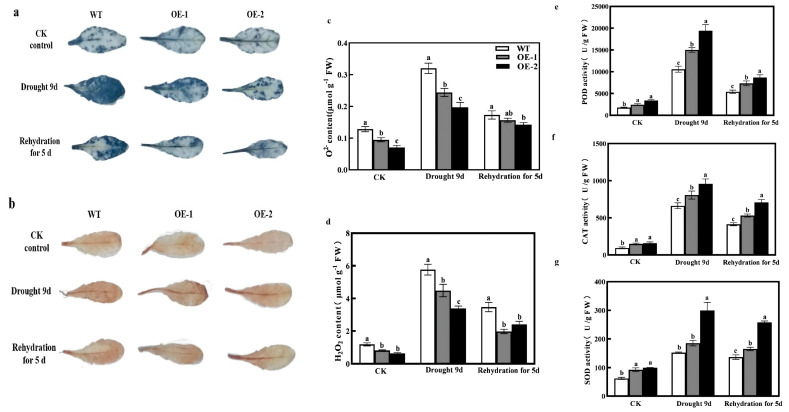
Overexpression of *CmXTH11* gene affects ROS accumulation and antioxidant activity in *Arabidopsis* leaves under drought stress. (**a**,**b**) NBT and DAB staining: The staining of leaves with NBT and DAB reveals the accumulation of O^2−^ and H_2_O_2_ in WT and *CmXTH11*-OE *Arabidopsis* plants under normal conditions, drought stress for 9 days, and after 5 days of rewatering. (**c**,**d**) ROS content: The quantification of O^2−^ and H_2_O_2_; (**e**–**g**) antioxidant enzyme activity: The activities of POD (**e**), CAT (**f**), and SOD (**g**) measured in leaves of WT and transgenic lines. Note: Data in the same column with different letters indicate significant differences (*p* < 0.05).

To investigate the role of *CmXTH11* in ROS scavenging under drought stress, NBT and DAB staining methods were used to detect the accumulation of O^2−^ and H_2_O_2_ in the leaves of WT and *CmXTH11*-OE *Arabidopsis* plants (Figure 8a,b). Under normal conditions, no significant difference in staining was observed between WT and transgenic plants. However, after stress treatment, staining intensity increased in both groups, with the *CmXTH11*-OE plants showing lighter staining compared to WT, indicating lower levels of ROS accumulation. Under normal growth conditions, the content of O^2−^ and H_2_O_2_ in WT leaves was higher than in *CmXTH11*-OE plants, although the differences were not substantial. Following stress treatment, ROS increased in both genotypes, but the accumulation was significantly higher in WT compared to the overexpressing lines (Figure 8c,d). The WT line exhibited increases in O_2_ and H_2_O_2_ levels of 31.15% and 62.43%, and 28.60% and 70.58%, compared to the OE-1 and OE-2 lines, respectively.

Additionally, the enzyme activities of SOD, CAT, and POD increased significantly under drought stress in all genotypes, with the transgenic plants showing higher enzyme activities than WT (Figure 8e–g). The activities of SOD, POD, and CAT in the OE-2 line increased by 95.86%, 44.71%, and 83.60%, respectively, compared to the WT line. After 5 days of rewatering, the activities of SOD, CAT, and POD decreased in all plants, but the enzyme activities in the transgenic lines remained higher than those in WT plants. These results suggest that overexpression of *CmXTH11* in *Arabidopsis* promotes the accumulation of antioxidant substances and enhances antioxidant enzyme activities, thereby improving the plant’s tolerance to drought stress.

### 2.6. Effects of Overexpression of CmXTH11 Gene against Drought Stress on Stomatal Characteristics

**Figure 9:**
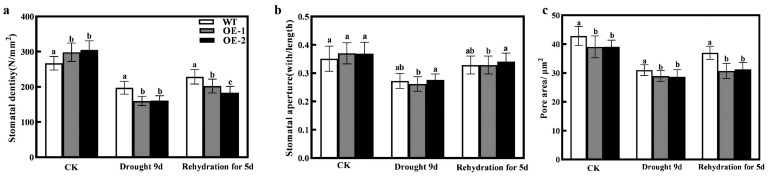
Impact of overexpressing *CmXTH11* gene on stomatal characteristics of *Arabidopsis* under drought stress. (**a**–**c**): (**a**) Stomatal density (**b**), stomatal aperture, and (**c**) pore area of WT and *CmXTH11* transgenic *Arabidopsis* leaves under normal growth conditions, after 9 days of drought stress, and after 5 days of rewatering. Note: Data in the same column with different letters indicate significant differences (*p* < 0.05).

Plants rapidly respond to external water availability by regulating changes in stomatal openings on the leaf surfaces. To investigate this response, we observed the stomatal phenotypes of WT and transgenic plants under different treatments. As shown in Figure 9a, stomatal density decreased in all three genotypes as drought severity increased. After 9 days of drought stress, stomatal density significantly decreased by 25.94%, 46.64%, and 47.54% in WT, OE1, and OE2, respectively, compared to the control (CK). Additionally, after 9 days of drought, the pore area in the WT line increased by 7.16% and 8.09% compared to the OE-1 and OE-2 lines, respectively (Figure 9c). After 5 days of rewatering, stomatal density and area increased in all three genotypes, and the WT line remained higher than the OE lines. There were no significant differences in stomatal aperture between WT and the transgenic lines under normal conditions, drought stress, or after rewatering (Figure 9b).

## 3. Discussion

Xyloglucan endotransglucosylase/hydrolases (*XTH*s) constitute a large gene family within the GH16 family of glycoside hydrolases. They are characterized by a conserved catalytic motif, DEIDFEFLG, which includes essential amino acid residues involved in catalytic reactions [21]. This study focused on the *CmXTH11* gene, selected based on previous experimental data from our lab, to investigate its functional role.

Sequence alignment of *CmXTH11* with homologous proteins from various plants revealed the highest homology with cucumber (*Cucumis sativus*), retaining the conserved catalytic motif DEIDFEFLG associated with XET/XTH activity (Figure 3a). Tertiary structure prediction confirmed this (Figure 1d). The expression of XTH genes exhibits tissue and organ-specific expression patterns. For instance, *AtXTH9* is specifically expressed in the stem tips, and its mutant *XTH9* displays a dwarf phenotype [22]. Similarly, *AtXTH17*, *AtXTH18*, *AtXTH19*, and *AtXTH20* are expressed in roots [23]. Our analysis of the tissue-specific expression of *CmXTH11* in roots, stems, and leaves of the melon variety “Emerald” indicates that *CmXTH11* is expressed in all three tissues, with the highest expression in the roots, followed by leaves and stems, showing a trend of roots > leaves > stems.

At the transcriptional level, *XTH* gene expression is regulated by plant hormones and environmental factors [24]. Research has shown that treatment with GA3 can induce the upregulation of *AtXTH21* expression in *Arabidopsis* seedlings [25]. The *SlXTH1* gene in tomato is primarily expressed in growing tissue cells, with auxin application leading to its upregulation in the hypocotyl [26]. *AtXTH19* and *AtXTH23* are induced by salt stress through the brassinosteroid signaling pathway transcription factor BES1 and play a role in lateral root development [27]. In this study, different concentrations of ABA, BR, GA3, and IAA were sprayed on melon seedlings, resulting in continuous changes in CmXTH11 expression within 24 h after treatment (Figure 2b).

Cell wall remodeling during plant growth involves changes in cell volume and number, with xyloglucan breakdown and regeneration being crucial [28]. In *Arabidopsis*, the overexpression of *AtXTH22* promotes cell division, elongation, and primary root growth [29]. The overexpression lines (OE) of *CmXTH11* exhibited significantly greater root lengths compared to wild-type (WT) plants (Figure 5). The number of bolting stems was notably higher in OE plants compared to WT (Figure 4c). Similarly, Liu et al. (2007) found that *AtXTH21*, by altering xyloglucan content and cellulose deposition, participates in root growth and promotes primary root elongation [25]. Fry et al. (1998) and Nishitani et al. (1995) have pointed out that *XTH*s play a major role in loosening cell walls and promoting cell elongation [30,31]. Increased catalysis of xyloglucan in hemicelluloses due to overexpression of *XTH* genes enhances cell expansion, regulates cell wall relaxation and elongation, resulting in increased root branching, primary root length, and bolting frequency.

Plant roots are the first organs to sense changes in the external environment, playing a crucial regulatory role in responding to stresses. A robust and elongated root system helps enhance plant drought resistance [32,33]. Our observations indicate that transgenic lines exhibited significantly greater root lengths and numbers of lateral roots compared to WT plants. Under osmotic stress from mannitol treatment, both WT and transgenic lines experienced inhibition of germination rate and root length. However, transgenic plants showed significantly higher germination rates and root lengths under varying concentrations of mannitol compared to WT plants (Figure 6), indicating that overexpression of the *XTH* gene promotes root growth and enhances osmotic stress tolerance in transgenic *Arabidopsis*.

After 9 days of drought stress, WT plants exhibited nearly complete wilting of leaves, while OE lines displayed only slight shrinkage. Following 5 days of rewatering, all *Arabidopsis* plants exhibited varying degrees of recovery, with the transgenic lines showing more pronounced recovery. This is likely due to the more developed root systems in the transgenic lines, which enhance water uptake capacity and enable them to cope more effectively with drought stress.

Reactive oxygen species (ROS) play a significant role in stress responses in plants [34]. Drought stress induces ROS generation and accumulation, leading to lipid peroxidation and cellular oxidative damage [35,36]. MDA content and electrolyte leakage serve as indicators of damage under water stress [37,38]. After 9 days of drought stress, both transgenic and WT plants exhibited increased MDA content and electrolyte leakage, but levels were significantly lower in OE1 and OE2 lines compared to WT (Figure 7b,c). After rewatering, the electrolyte leakage and cell damage in transgenic plants remained significantly lower than in WT, reflecting a similar trend in relative water content. Drought stress increased O^2−^ and H_2_O_2_ levels in all *Arabidopsis* lines, but OE1 and OE2 lines accumulated significantly less of these ROS compared to WT (Figure 8a,b). NBT and DAB staining results corroborated the changes in O^2−^ and H_2_O_2_ levels. Antioxidant defense systems and redox homeostasis are critical for balancing ROS generation and maintenance, alleviating oxidative stress under various environmental conditions [39]. SOD dismutates O^2−^ to H_2_O_2_, which is further decomposed by CAT and POD to protect cells from oxidative damage [40]. Our study demonstrates that under 9 days of drought stress, SOD, CAT, and POD activities were significantly higher in *CmXTH11* overexpression lines compared to WT. After 5 days of rewatering, while both WT and OE plants showed recovery, SOD, CAT, and POD activities remained higher in OE lines (Figure 8c–g). Previous research has shown that overexpression of *GhXTH22* in Arabidopsis significantly reduced the content of harmful substances (H_2_O_2_ and MDA), but significantly increased antioxidant enzyme activity (CAT and POD) [41]. Shi et al. reported that when exposed to 4 °C stress, plants overexpressing *AtXTH21* exhibited significantly lower concentrations of H_2_O_2_, O^2−^, and MDA, along with higher activities of SOD, CAT, and POD compared to wild-type plants [42]. It is suggested that overexpression of *CmXTH11* can effectively regulate the accumulation of O^2−^ and H_2_O_2_ in *Arabidopsis* leaves under drought stress, enhance antioxidant enzyme activity, mitigate the damage of reactive oxygen species to membrane lipids, maintain the integrity of the cell membrane, and thereby improve drought tolerance in plants.

Plants dynamically regulate stomatal aperture in response to environmental changes [43]. Stomatal conductance is controlled by both stomatal density and size, with many plants adjusting their stomatal openings to adapt to environmental conditions [44,45]. In drought-resistant plants, smaller stomatal density and pore size help reduce transpiration and improve water use efficiency. Under drought stress, *HvXTH1* transgenic plants exhibited larger stomatal apertures compared to WT [46]. Choi et al. found that overexpression of *CaXTH3* significantly enhanced tomato drought tolerance by affecting stomatal closure [15]. Similarly, in our study, while stomatal aperture was not significantly different among lines, drought stress and rewatering affected stomatal density and area. WT plants had significantly higher stomatal density and area compared to transgenic lines (Figure 9). This suggests that *CmXTH* may influence stomatal closure and opening under drought stress by modulating cell wall flexibility and elasticity, in turn, enhances the plant’s drought resistance.

## 4. Materials and Methods

### 4.1. Plant Materials

Melon seeds of the variety “Emerald” (*Cucumis melo* L.) were purchased from Shandong Zibo Hefeng Seed Industry Technology Co. (Zibo, China). The seeds were soaked in distilled water for 8 h and then germinated in the dark for 48 h. Following germination, the seeds were sown in 10 cm × 10 cm pots and grown in a controlled climate chamber with a light intensity of 150 W/m^2^, a day/night temperature of 25 °C/20 °C, and a photoperiod of 12 h light/12 h dark. Cotyledons were sampled for *CmXTH11* gene cloning and tissue-specific expression analysis. Samples were immediately frozen in liquid nitrogen and stored at −80 °C until use. The wild type of *Arabidopsis* is the Columbia type (Col-0), which was maintained in the laboratory.

### 4.2. CmXTH11 Gene Structure and Expression Analysis

When melon seedlings reached the three-leaf stage, they were sprayed with 150 mg/L abscisic acid (ABA), 200 mg/L gibberellin (GA3), 0.02 mg/L methyl jasmonate (MeJA), and 30 mg/L indole-3-acetic acid (IAA) until droplets fell down. The control group was sprayed with distilled water only. The molecular weight, isoelectric point, and hydrophobicity of the *CmXTH11* protein were predicted using the ExPASy Proteomics Server (http://web.expasy.org/protparam/ (accessed on 8 September 2024)) [47]. Its transmembrane structure and signal peptide were predicted using the SignalP 4.1 server (https://services.healthtech.dtu.dk/services/SignalP-4.1/ (accessed on 8 September 2024)) [48] and TMHMM-2.0 (https://services.healthtech.dtu.dk/services/TMHMM-2.0/ (accessed on 8 September 2024)) [49]. Using the SWISS-MODEL website (https://swissmodel.expasy.org/ (accessed on 8 September 2024)) to predict the tertiary structure of the encoded amino acids [50], the amino acid sequence of *CmXTH11* was aligned with homologous sequences from six plants (bitter melon (*Momordica charantia*), wax gourd (*Benincasa hispida*), pumpkin (*Cucurbita moschata*), *Arabidopsis thaliana*, cucumber (*Cucumis sativus*), and tomato (*Solanum lycopersicum*)) using MEGA7 software 11.0.10 to construct a multiple sequence alignment and phylogenetic tree.

### 4.3. Generation of Transgenic Arabidopsis Plants Overexpressing CmXTH11

Total RNA was extracted from melon cotyledons using the SPARKeasy Plant RNA Kit (SparkJade, Jinan, China). Complementary DNA (cDNA) was synthesized from RNA using the RevertAid First Strand cDNA Synthesis Kit (Thermo Scientific, Waltham, MA, USA). The coding region of *CmXTH11* was obtained from melon cotyledons. Primers were designed for gene cloning based on the principle of homologous recombination, incorporating NcoІ and BstEII restriction sites and 15 bp homology fragments flanking the *CmXTH11*-specific primers. The primer sequences are listed in Table S1. Double digestion was performed using NcoІ and BstEII restriction enzymes. The target fragment was ligated with the pCAMBIA1303 vector fragment using a homologous recombination enzyme according to the manufacturer’s protocol, resulting in the insertion of the *CmXTH11* open reading frame (ORF) into the pCAMBIA1303 vector downstream of the 35S promoter, thus constructing the expression vector pCAMBIA1303-*CmXTH11*. The recombinant plasmid was introduced into *Agrobacterium tumefaciens* GV3101. *Arabidopsis thaliana ecotype Columbia* (Col-0) was transformed using the floral dip method [51], and T3 homozygous transgenic lines (OE1 and OE2) were obtained for further experiments.

### 4.4. Experimental Treatments

Wild-type and two T3 homozygous transgenic *Arabidopsis* seeds were sown on 1/2 MS medium. The seeds were cold-stratified at 4 °C for 2–3 days. The plates were then placed in a growth chamber with a 16 h light/8 h dark cycle at 22 °C. After vertical cultivation for 10 days, photographs were taken and statistical analysis was performed to assess the root growth conditions of the transgenic lines and wild-type *Arabidopsis*.

To investigate the effect of drought stress on seed germination and growth in *Arabidopsis*, seeds were sown on 1/2 MS medium. After 5 days, seedlings were transferred to 1/2 MS solid medium containing 100, 200, or 300 mmol/L mannitol, with 0 mmol/L mannitol as the control. This included two experiments: (1) photographic documentation of phenotypes included both root length and germination; (2) measuring root length after the plants were grown vertically for 10 days; (3) calculating the germination rate after 10 days of growth. The number of green cotyledons was assessed 12 days later.

For soil drought stress, wild-type and *CmXTH11* transgenic *Arabidopsis* seeds were sown in nutrient soil, maintaining soil moisture. After 30 days, healthy and similarly growing plants were selected. Soil drought was simulated by withholding water, and relevant parameters were measured after 9 days of drought and 5 days of rehydration.

### 4.5. Analysis of Plasma Membrane Permeability and Malondialdehyde (MDA) Content

Relative water content (RWC) and relative electrolyte conductivity (REC) were measured using the methods of Xu et al. (2020) [52]. Malondialdehyde (MDA) content was determined using a commercial kit (Solarbio Biotechnology Co., Ltd. Beijing, China).

### 4.6. Determination of H_2_O_2_/O^2−^ Content and DAB/NBT Staining

Hydrogen peroxide (H_2_O_2_) and superoxide anion radical (O^2−^) contents were measured using H_2_O_2_ and O^2−^ content detection kits (Solarbio, Beijing, China) and quantified with a spectrophotometer. Reactive oxygen species (ROS) distribution in leaf tissues was visualized using histochemical staining with 3,3-diaminobenzidine (DAB) and nitroblue tetrazolium (NBT) [53]. Activities of superoxide dismutase (SOD), peroxidase (POD), and catalase (CAT) were measured using the methods described by Zhang et al. (2005) and Khan et al. (2021) [54,55].

### 4.7. Measurement of Stomatal Apertures

For each treatment, three seedlings were randomly selected for stomatal preparations. Leaf samples were adhered to transparent tape, lightly pressed to ensure adhesion, then the leaf tissue was scraped off with a blade. The preparations were mounted on glass slides, and stomata were observed under a microscope (Olympus BX63, Olympus LS, Tokyo, Japan). Stomatal density was measured at 20× magnification, while stomatal size was measured at 40× magnification. Stomatal density, pore length, and width were quantified using ImageJ software (Version 1.54k, National Institutes of Health, Bethesda, MD, USA). Pore area was calculated using the following formula: π × (pore length/2) × (pore width/2). Stomatal aperture = width/length.

### 4.8. RT-qPCR Analysis

Total RNA from leaf tissues was extracted using the SPARKeasy Plant RNA Kit (SparkJade, Jinan, China) following the manufacturer’s protocol. cDNA was synthesized from the RNA using a reverse transcription kit (Cofitt, Kowloon, Hong Kong) according to the manufacturer’s instructions. qPCR was performed using the 2×qPCR SmArt Mix (SYBR Green) kit (Dr. Di, Shanghai, China) in a 20 μL reaction volume. Relative gene expression levels were analyzed using the 2^−ΔΔCt^ method [56]. Primers were designed using Primer Premier 5.0, and the primer sequences are listed in Table S1.

### 4.9. Statistical Analysis

All experiments were independently repeated at least three times, and results are presented as mean ± standard deviation. One-way analysis of variance (ANOVA) was performed using IBM SPSS Statistics 27 with the post hoc test of Duncan’s method, and differences were considered statistically significant at *p* < 0.05 or *p* < 0.01. Graphs were generated using GraphPad Prism 9.5 software (GraphPad Software, San Diego, CA, USA).

## 5. Conclusions

Xyloglucan endotransglucosylase/hydrolase (*XTH*) plays an important role in cell wall modification by cleaving and recombining xyloglucan, participating in various physiological processes. This study found that the heterologous expression of the *CmXTH11* gene significantly enhanced the drought tolerance of transgenic *Arabidopsis*, demonstrating superior root elongation and germination rates. This finding suggests that *CmXTH11* may serve as an effective target gene for improving stress resistance in crops. Additionally, the higher antioxidant enzyme activity in transgenic plants, along with the reduced stomatal area, may help minimize transpiration losses under drought conditions. These results not only provide new insights into the mechanisms of plant responses to adversity but also offer a potential application basis for genetic engineering strategies in agricultural crops, carrying significant agricultural and economic implications.

## Figures and Tables

**Table 1:** *XTH* protein sequences from six plant species.

Name	Protein Sequence Number
*Cucumis melo*	XP_008466018.1
*Cucumis sativus*	CCH26637.1
*Benincasa hispida*	XP_038887272.1
*Cucurbita moschata*	XP_022963979.1
*Momordica charantia*	XP_022156021.1
*Arabidopsis thaliana*	NP_174496.1
*Solanum lycopersicum*	AAS46240.1

## Data Availability

Data are contained within the article.

## Supplementary Materials

The following supporting information can be downloaded at: https://www.mdpi.com/article/10.3390/ijms252011031/s1.
